# Case of Necrosis and Expulsion of the Os Hyoides

**Published:** 1848-10

**Authors:** 


*Case of Necrosis and Expulsion of the Os Hyoides.*—Miss B. 41 years of age, deformed from rickets, was subjected to medical treatment for a swelling on the upper part of the neck, below the ear. She was not much benefitted, and the tumor remained for a period of about five years. At length she began to be troubled with cough, and expectorated much tough frothy mucus, which was often streaked with blood. After a while portions of false membrane were also ejected; she lost her voice, and the respiration became difficult; she suffered from hectic fever, &c. One day, in a fit of coughing, she felt a hard body in the throat, which excited vomiting also, when a bone was expelled; after which her cough and difficulty of breathing abated, her voice returned in a few days, and she every day regained health and strength. On examination, the bone was found to be the hyoid bone, quite carious on its internal surface, but smooth on its external surface. The anterior and upper part of the neck, instead of projecting as usual, appears unnaturally flat and broad, but she has completely recovered the power of swallowing, which was difficult for a considerable time before the expulsion of the os hyoides.—*Bulletin de l'Academie Royale de Médecine.*

